# Anatomical results and complications after silicone oil removal

**Published:** 2017

**Authors:** Daniel Constantin Brănişteanu, Andreea Moraru, Andrei Bîlha

**Affiliations:** * Ophthalmology Department, “Gr. T. Popa” University of Medicine and Pharmacy, Iasi, Romania; ** ”RETINA CENTER” Eye Clinic, Iasi, Romania; *** Ophthalmology Clinic, “N. Oblu” Clinical Emergency Hospital, Iasi, Romania; **** Ophthalmology Clinic, “Sf. Spiridon” Clinical Emergency Hospital, Iasi, Romania; ***** Physiology Department, “Gr. T. Popa” University of Medicine and Pharmacy, Iasi, Romania

**Keywords:** retinal detachment, 23G vitrectomy, silicone oil removal

## Abstract

**The aim** of the report was to evaluate anatomical results and intra and postoperative complications after silicone oil (SIO) removal.

****Methods**:**

Retrospective, interventional study evaluating consecutive cases with ambulatory SIO removal after vitrectomy for complex retinal detachments. The anatomical result was the main followed parameter. Intra and postoperative complications and also intraocular pressure changes were evaluated. Cases were followed-up for at least 12 months.

****Results**:**

A total of 98 consecutive cases were reviewed. The main duration of oil endotamponade was 5.46 months (3–16 months). In 15 cases (15.30%) signs of SIO emulsification were noted at the time of removal. A stable anatomical result after SIO removal was obtained in 94 out of 98 cases (95.91%). Retinal detachment recurrence appeared in first month postoperatively (1 case) and between the 3rd and 4th month postoperatively (3 cases). Main indications for 5000cs SIO endotamponade during ambulatory 23G vitrectomy were represented by proliferative vitreoretinopathy (PVR) (78 cases - 79.59%), proliferative diabetic retinopathy (17 cases - 17.34%) and giant retinal tears (3 cases - 3.06%). 29 eyes (29.59%) were pseudophakic at primary surgery. However, most phakic eyes showed cataract appearance and progression during SIO endotamponade and also after SIO removal. Intraocular pressure significantly decreased after SIO removal with the occurrence of various choroidal detachments in 8 cases (8.16%), resolving spontaneously within the first week postoperatively.

****Conclusions**:**

In our experience, the retinal detachment recurrence rate after SIO removal was 4.08%. This promising anatomical result confirms the need for an accurate primary surgery and also for a safe moment of SIO removal according to the severity of primary pathology.

## Introduction

Complex retinal detachments occur in the presence of proliferative vitreoretinopathy (PVR), proliferative diabetic retinopathy, giant retinal tears, ocular trauma and others, and often require silicone oil (SIO) endotamponade after vitrectomy [**1**]. 

Since its first use [**2**], SIO endotamponade quickly evolved and is nowadays widely used in vitreoretinal surgery with different purposes. It is known to be a very effective long-term solution in cases that underwent pars plana vitrectomy to stabilize complex retinal detachments. Still, despite constant improvement, the relative tolerability and safety profile obliges to SIO removal as soon as the retina has been stabilized or SIO related complications appear [**3**]. 

Although refractive change during endotamponade is a constant disturbing feature, more serious complications are linked to persistence of SIO as intraocular endotamponade agent: cataract, keratopathy, rubeosis iridis, macular pucker, cystoid macular edema, optic nerve atrophy and migration of the SIO in anterior chamber or even further to the brain [**4**]. PVR reproliferation and retinal redetachment are also serious adverse events of intraocular SIO endotamponade. Angle closure due to oil-induced pupillary block usually occurs in aphakic eyes if a prophylactic six o'clock iridectomy is not performed.

Complications like glaucoma, inverse hypopyon in anterior chamber, corneal and retinal toxicity are related to the emulsification of SIO. The emulsification time largely varies among individuals and is mostly related to endotamponade duration but also to viscosity and molecular weight. One study showed that first signs of emulsification largely varied from 5 to 24 months after initial surgery. As from a statistical point of view, the emulsification process arises at an average time of 13 months after surgery; therefore, it might be reasonable to extend the SIO removal moment, in selected cases, up to one year in order to obtain stable retinal status [**5**]. Higher-viscosity SIO is considered to have a lower tendency to emulsify than low-viscosity SIO, thus offering the possibility to minimize the percentage of complications due to oil emulsification, but literature results remain controversial [**6**].

The SIO endotamponade has to be maintained as long as it is necessary according to physician’s judgement. An early removal would be advisable but it might also compromise the retinal attachment [**7**]. Regular follow-ups are important in order to assess both the anatomical and functional status of the eye and also SIO related complications. While the exact moment of removal is still under debate, most authors agree that it has to be removed within the first year after surgery to minimize significant risks. 

One of the most important complication after SIO removal is the retinal detachment recurrence. In literature, the incidence largely varies between 3.5 and 34%. Patient selection, surgical techniques and time before SIO removal [**8**] are considered significant factors influencing the result. Primary disease is also very important and influences the rate of reported recurrence of retinal detachment after SIO removal. The rate seems to be higher in eyes with severe PVR and complicated trauma and lower in proliferative diabetic retinopathy and giant retinal tears [**9**]. 

The purpose of this study was to evaluate the anatomical results after silicone oil removal in a personal series, as well as the complications encountered intra and postoperatively.

## Material and Methods

This is a retrospective, interventional study, evaluating consecutive cases that underwent SIO removal after primary 23G vitrectomy for complex retinal detachments. All cases were operated by the same surgeon using local anesthesia. Alcon CONSTELLATION® Vision System was used at both primary vitrectomy and later on to actively remove the silicone oil. Oxane 5700 silicone oil was used for endotamponade in all cases. 

The anatomical result was the main followed parameter. Intra and postoperative complications and also intraocular pressure changes were evaluated. Cases were followed-up for at least 12 months.

## Results

A total of consecutive 98 eyes from 98 patients were reviewed. The mean age of patients was 53.7 years (28-72 years). The main indications for SIO endotamponade during ambulatory 23G vitrectomy were represented by proliferative vitreoretinopathy (PVR) (78 cases - 79.59%), proliferative diabetic retinopathy (17 cases - 17.34%) and giant retinal tears (3 cases - 3.06%). 29 eyes (29.59%) were pseudophakic at primary surgery. 

The main duration of oil endotamponade was 5.46 months (3–16 months). 51 out of 98 cases (52.04%) had the SIO removed within the first 6 months, 43 (43.87%) within the next 6 months and only 4 cases (4.08%) required maintaining the SIO endotamponade for more than 1 year up to removal moment. Signs of SIO emulsification were noted in 21 cases (21.42%), most of them (17 cases – 80.95%) after 6 months of endotamponade. 

A significant number of eyes with no preexisting glaucoma developed increased intraocular pressure over 21 mmHg during SIO endotamponade (52 out of 98 cases - 53.06%) and required topical lowering medication. After SIO removal, the intraocular pressure decreased gradually and only 16 out of 98 eyes (16.32%) still required medication at 12 months follow-up.

**Fig. 1:**
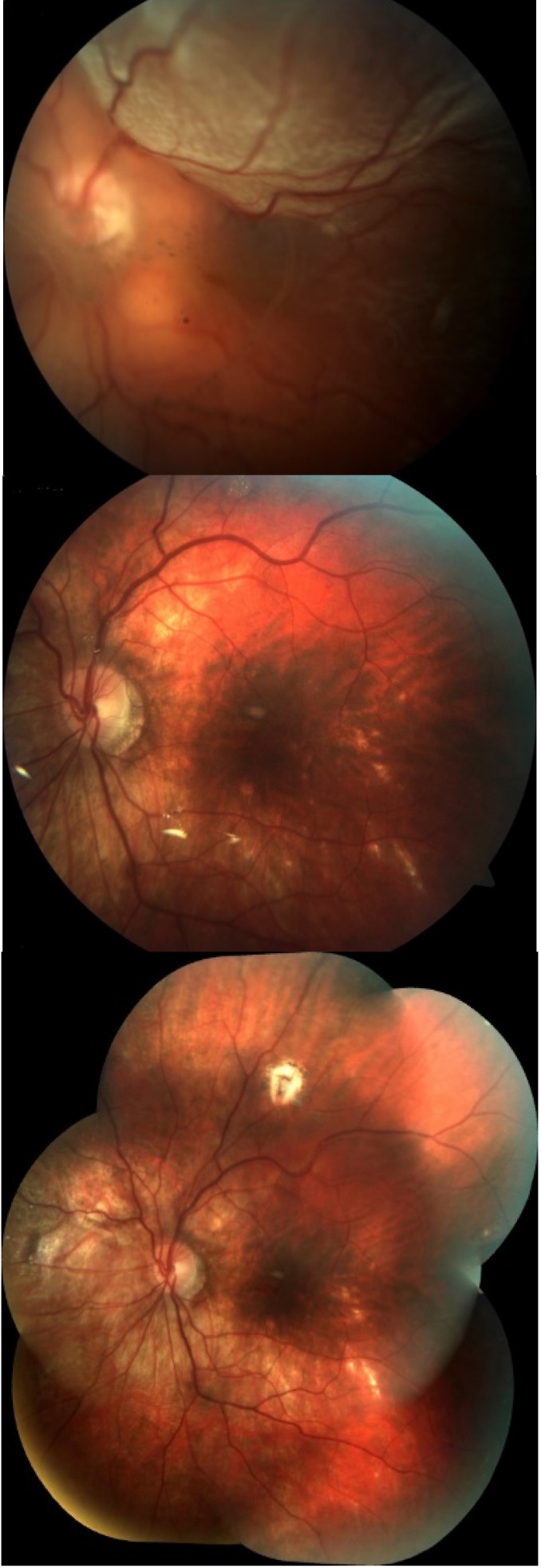
Retinal detachment related to a large peripheral tear. Left image: before surgery; Middle image: during SIO endotamponade; Right image: 12 months after SIO removal

**Fig. 2:**
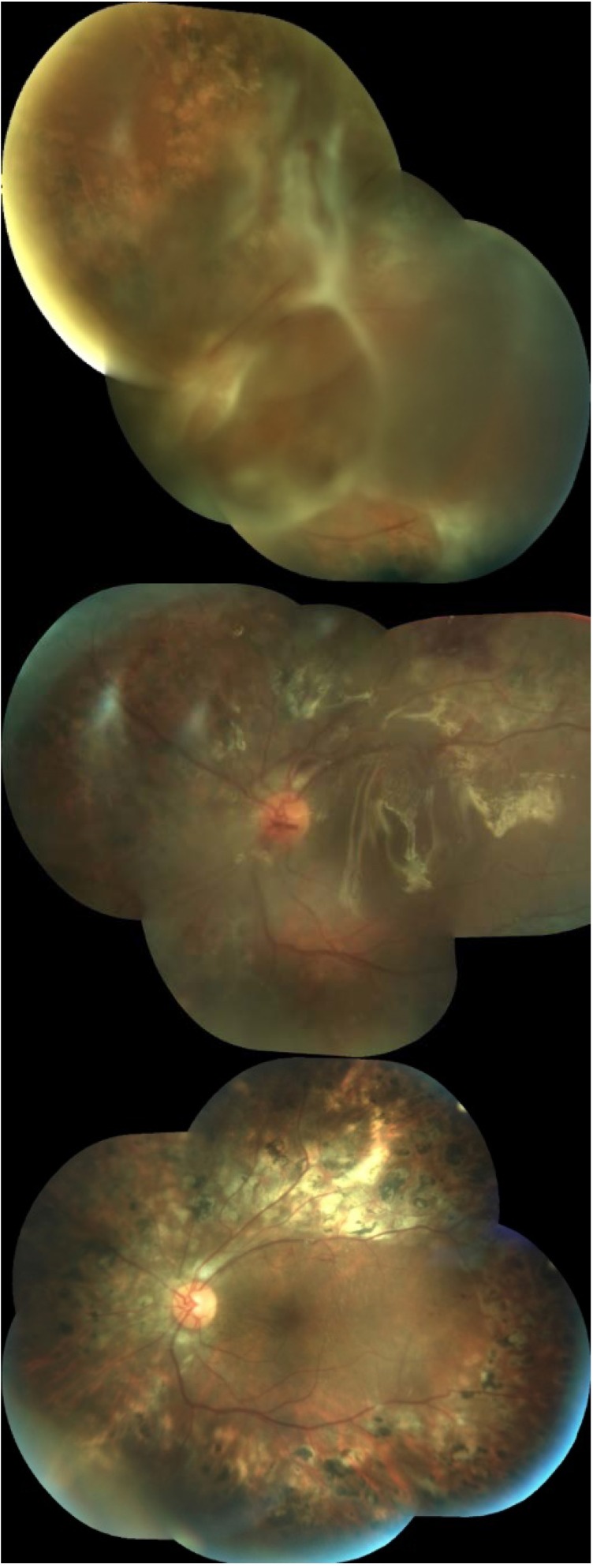
Mixed retinal detachment in proliferative diabetic retinopathy. Left image: before surgery; Middle image: during SIO endotamponade; Right image: 12 months after SIO removal

Anatomical success, defined as a stable attached retina at 12 months follow-up after SIO removal, was achieved in 94 out of 98 cases (95.91%) (**Fig. 1** and **2**). Visual acuity increased with at least 2 lines in all cases.

Retinal detachment recurrence occurred in 4 cases, one in the first month postoperatively and the other 3 cases between the 3rd and 4th month. One of the patients had diabetic retinopathy, and the other 3 were operated for proliferative vitreoretinopathy in the presence of high myopia. 

No intraoperative complications were encountered at the time of SIO removal.

**Fig. 3:**
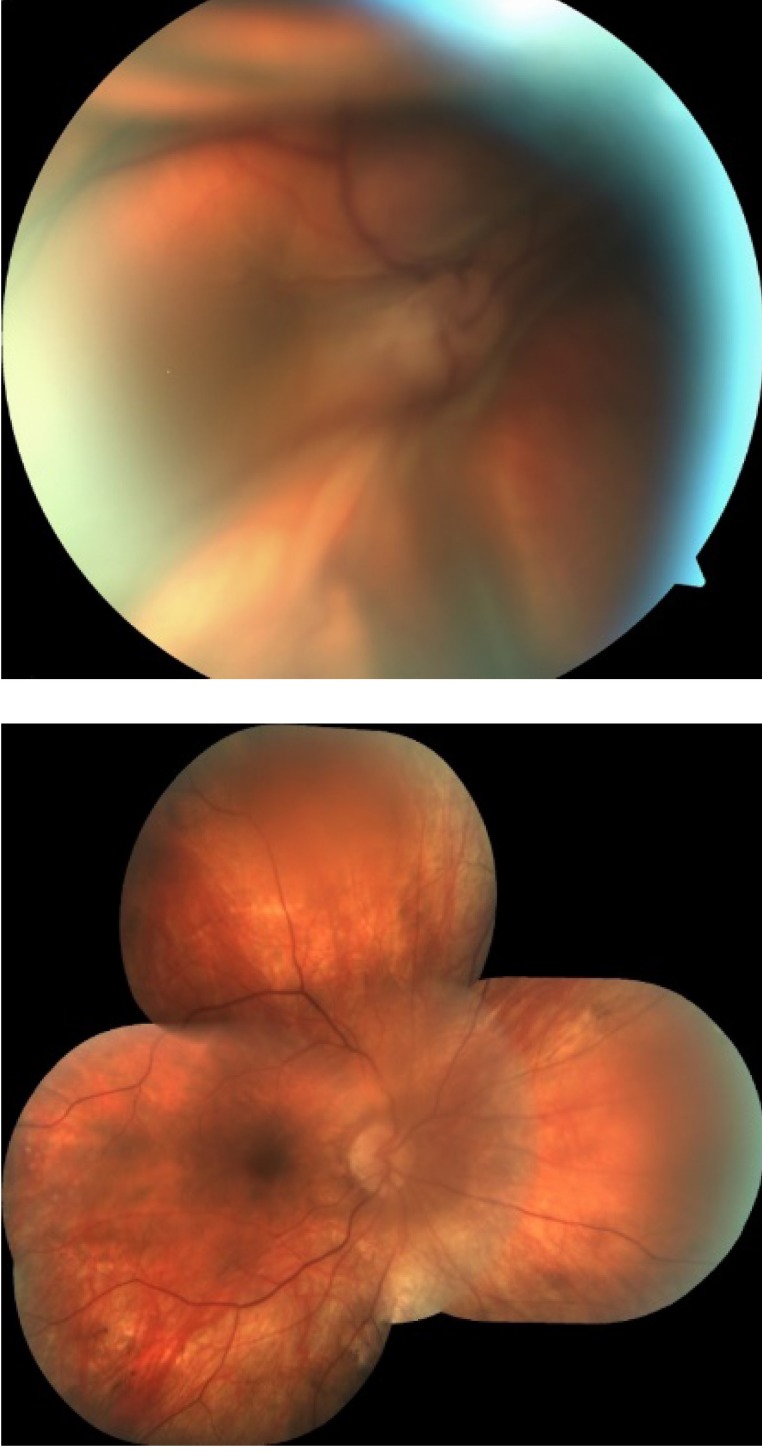
Massive choroidal detachment due to postoperative hypotony. Left image: At 24 hours after SIO removal; Right image: completely resolved spontaneously at 7 days postoperatively;

Transient hypotony affected 38 out of the 98 eyes (38.77%) next day after SIO removal and 8 eyes (8.16%) developed various choroidal detachments resolving spontaneously within the first week postoperatively (**Fig. 3**). 

Most phakic eyes showed cataract development and progression during both SIO endotamponade and after SIO removal. At 12 months follow-up, 47 out of 69 phakic patients (68.11%) required cataract surgery.

At last follow-up, 4 cases (4.08%) presented small residual asymptomatic silicone bubbles in the vitreous cavity. 

## Discussions

Literature data does not provide a precise timing for SIO removal. While early removal might impact the retinal stability, a late removal might be difficult because of associated SIO complications. A median 30 months endotamponade is responsible for significant ocular complications, such as optic atrophy (28%), corneal decompensation (12%), band keratopathy (8%) and rubeosis iridis (14%) [**10**,**11**]. 

One of the most fearful postoperative complication after SIO removal is the recurrence of retinal detachment. According to literature, statistically significant risk factors for redetachment are the quality of primary surgery (incomplete removal of the vitreous base, previous number of retinal surgeries, surgeon’s experience), the severity of initial PVR and vitreous hemorrhage appearance in the first 3 days after SIO removal. PVR is associated with the highest risk of retinal redetachment, whereas diabetic retinopathy, high myopia and giant retinal tears with a lower risk of redetachment [**9**,**12**]. Surprisingly, the technique of SIO removal and the duration of the SIO endotamponade were found of no prognostic significance concerning retinal redetachment by certain authors [**7**,**13**]. 

Retinal detachment recurrence rate of 4.08% achieved in our series is comparable to most optimistic data of 3.46% [**9**]. In order to avoid retinal redetachment, an accurate primary surgery is mandatory.

A common complication encountered during SIO removal is early hypotony. In our series, a transient hypotony was noted in 38 out of the 98 eyes (38.77%) next day after SIO removal. All 8 eyes (8.16%) that additionally developed choroidal detachment evolved favorably with spontaneous recovery within the first week postoperatively. Literature data points out that the incidence of postoperative hypotony after SIO removal varies between 25% and 40% [**14**]. Risk factors for early hypotony are: a longer axial length [**15**], infusion cannula retraction [**16**] and incorrect trocar insertion. Apparently, with a 23 G system there is a lower risk of hypotony as compared to other small G sutureless vitrectomies. Chen et al. pointed out that gaping, misalignment, and important variation in incision angle might have a negative impact on sutureless sclerotomy [**17**]. 

When removing SIO, it is recommended to be removed as complete as possible. Small silicone droplets can remain somehow attached to the trocar or on the inner surface of the retina and move freely within the vitreous cavity after that. Modern equipment can facilitate total removal and provide efficient active aspiration of high-density SIO even through small trocars.

The key for a lower rate of retinal detachment recurrence after SIO removal relies on both initial pathology severity and also on primary surgery quality. Careful removal of vitreous base and peeling of epiretinal membranes, sufficient laser retinopexy, use of relaxing retinotomies in advanced PVR and complete SIO filling are correlated with a lower incidence of retinal redetachment [**8**,**9**].

## Conclusions

In our experience the retinal detachment recurrence rate after SIO removal was 4.08%. This promising anatomical result confirms the need for an accurate primary surgery and also for choosing a safe moment for SIO removal according to the severity of primary pathology.
